# Supplementary material for: Knowledge-graph-based cell-cell communication inference for spatially resolved transcriptomic data with SpaTalk
Source: Nat Commun. 2022 Jul 30;13:4429. doi: 10.1038/s41467-022-32111-8 (PMC9338929; doi:10.1038/s41467-022-32111-8)
Supplement: Supplementary file 1 — Supplementary information [file 41467_2022_32111_MOESM1_ESM.pdf]

Supplementary Figures

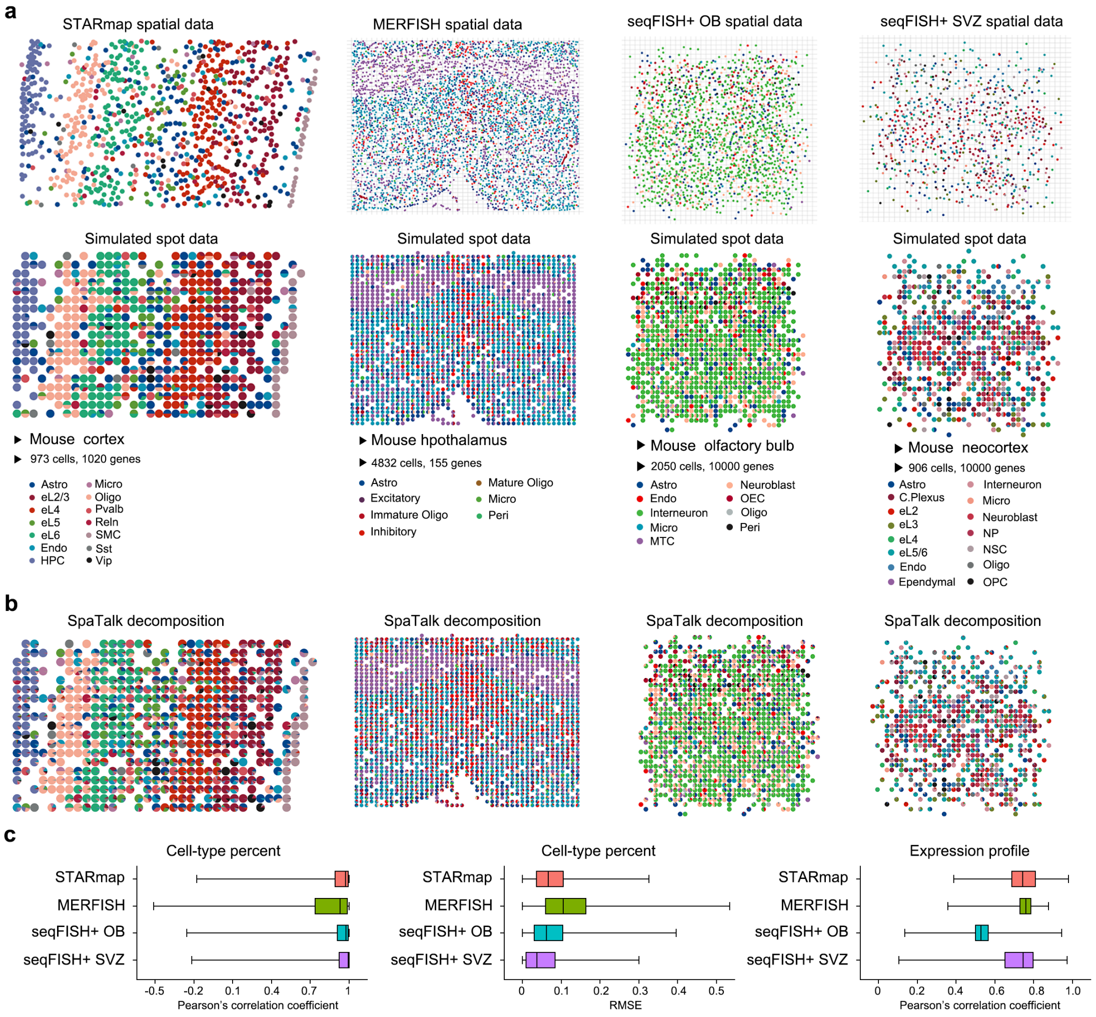

Fig. 1 Benchmarked datasets and cell-type decomposition by SpaTalk. a Selected three spatial technologies and four ST datasets at single-cell resolution, namely the STARmap mouse cortex, MERFISH mouse hypothalamus, seqFISH+ OB and SVZ datasets. **b** Cell-type decomposition by SpaTalk over the benchmarked datasets. **c** Performance of SpaTalk over the benchmarked datasets in cell-type decomposition. Pearson's correlation coefficient and RMSE were used to evaluate the predicted and real cell-type percent as well as the expression profile between the spot and the predicted optimal cellular combination for each simulated spot. For the boxplots (minima, 25th percentile, median, 75th percentile, and maxima) in terms of the STARmap, MERFISH, seqFISH+ OB, and seqFISH+ SVZ datasets, the numbers of data points are 509, 1,844, 986, and 626, respectively. OB, olfactory bulb; SVZ, sub-ventricular zone; RMSE, root mean square error.

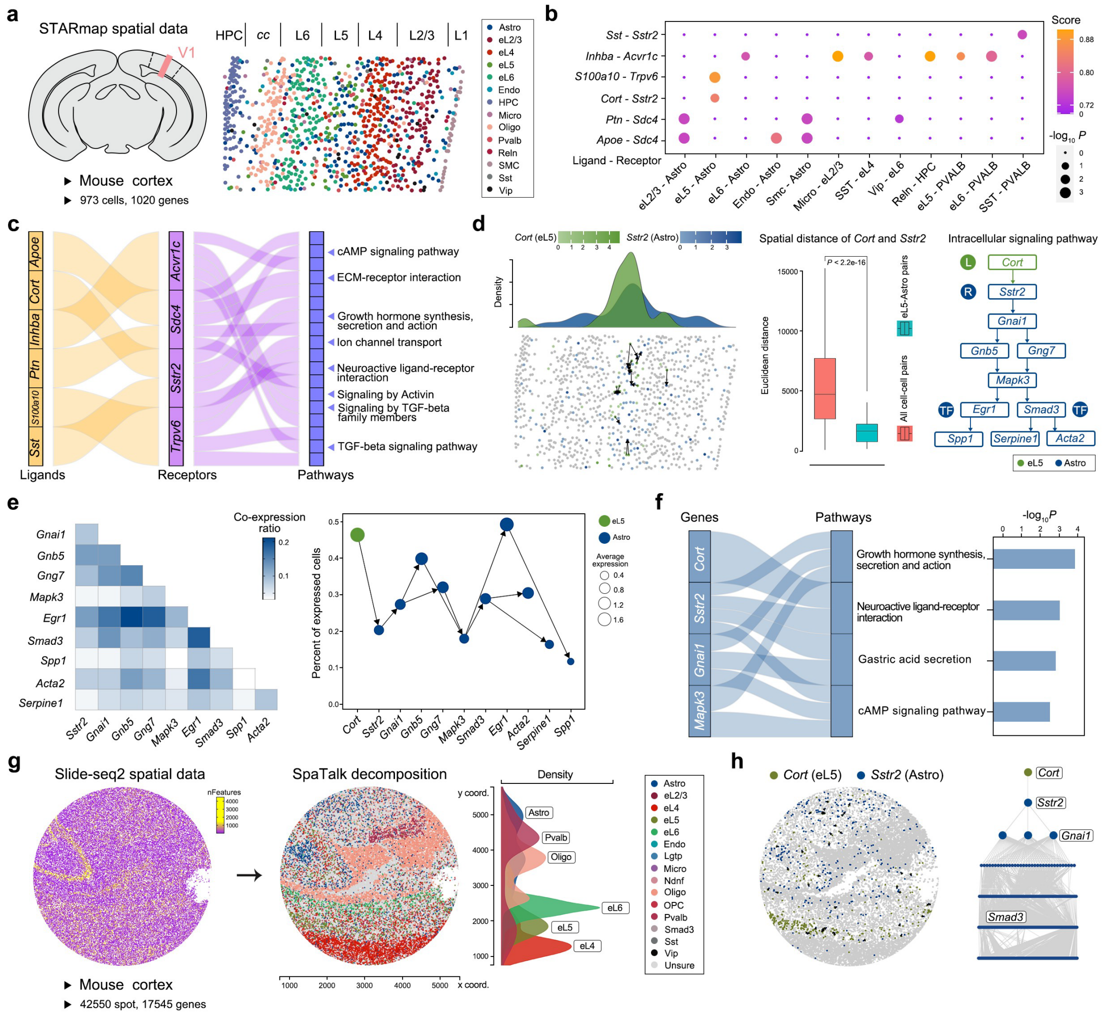

Fig. 2 Identification of spatial inhibitory signal transmission among neurons and non-neuronal cells. a STARmap single-cell ST dataset of the mouse visual cortex involving 973 cells and 1,020 genes. Astro, astrocytes; eL2/3, eL4, eL5, eL6, excitatory neuron subtypes; Endo, endothelial cells; HPC, hippocampus; Micro, microglia; Oligo, oligodendrocytes; SMC, smooth muscle cells; cc, corpus callosum. **b** Significantly enriched LRIs that mediate cell–cell communications among neurons and non-neuronal cells inferred by SpaTalk with $P < 0.05$. **c** Sanky plot of the associations among ligands, receptors, and biological processes or pathways in the KEGG and Reactome databases that mediate cell–cell communications in the central nervous system. **d** Spatial distribution and intra-cellular signaling pathways of the *Cort–Sstr2* pairs between the eL5 senders and Astro receivers. *P* values were calculated with the one-sided t-test. For the boxplots (minima, 25th percentile, median, 75th percentile, and maxima), the numbers of data points from left to right are 99,229 and 1,269, respectively. **e** Co-expression of target genes in receivers and the percentage of expressed cells for target genes. **f** Significantly enriched biological processes and pathways with the ligand-receptor-target genes using the two-sided Fisher exact test. **g** Slide-seq spot-based ST dataset of the mouse visual cortex involving 42,550 spots and 22,542 genes. OPC, oligodendrocyte progenitor cell. **h** Communications of eL5-

Astro mediated by the *Cort–Sstr2* interaction in space and the intra-cellular signal pathway inferred by SpaTalk over Slide-seq data.

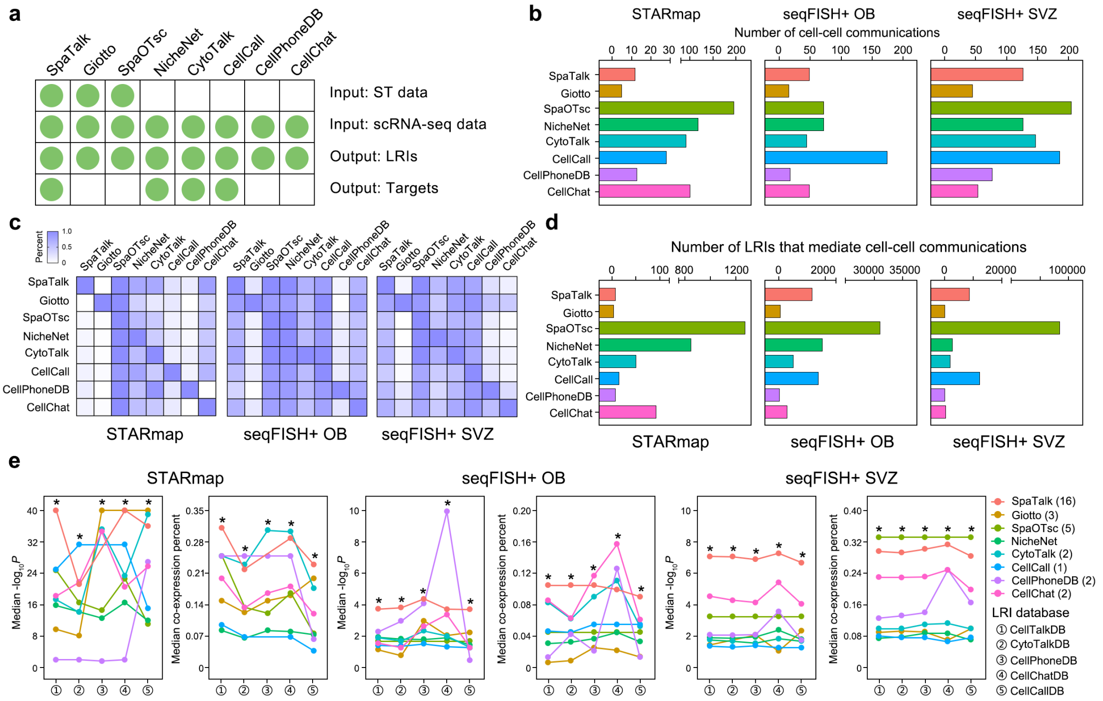

Fig. 3 Comparison of SpaTalk with other methods. a Selected representative cell-cell communication inference method, namely the Giotto, SpaOTsc, NicheNet, CytoTalk, CellCall, CellPhoneDB, and CellChat. Requirements of input data and the type of output data were shown. **b** Number of cell-cell communications inferred by different methods over the STARmap, seqFISH+ OB and SVZ ST data at single-cell resolution. **c** Percent of shared cell-cell communications between paired methods. For each row, the value means the number of overlapped cell-cell communication divided by the number of inferred cell-cell communications for this method. **d** Number of LRIs that mediate cell-cell communications inferred by benchmarked methods over the STARmap, seqFISH+ OB and SVZ ST datasets. **e** Comparison of SpaTalk with other methods over the different LRI databases. The asterisk represents the top-ranked method for each used LRI database.

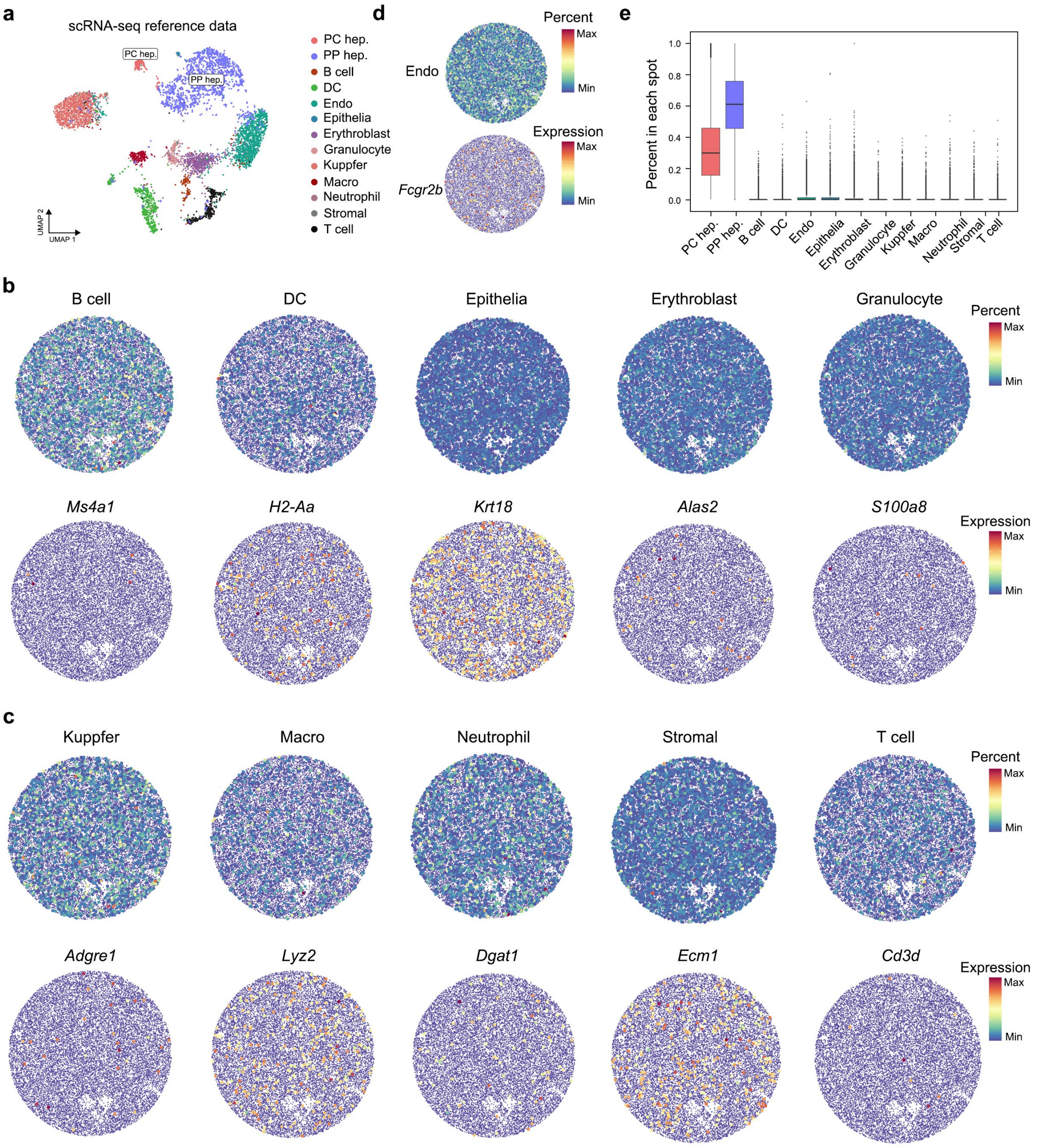

Fig. 4 Cell-type decomposition on the mouse liver ST data of Slide-seq. **a** Mouse liver scRNA-seq reference integrating the non-parenchymal cells from the mouse cell atlas (MCA) and the parenchymal hepatic cells from GSE125688, which contains 6,029 cells involving the major immune cells and the pericentral and periportal hepatocytes, etc. **b-d** Expression of known marker gene (up) and the percent (down) for Endo, B cell, DC, Epithelia, Erythroblast, granulocyte, Kupffer cell, Macro, neutrophil, stromal cell, and T cell. **e** Percent of cell types across 25,595 spots of Slide-seq data. For the boxplots (minima, 25th percentile, median, 75th percentile, and maxima), the numbers of data points for each box are 25,595. PC, pericentral; PP, periportal; Hep, hepatocytes; Endo, endothelial cells; DC, dendritic cells; Macro, macrophages.

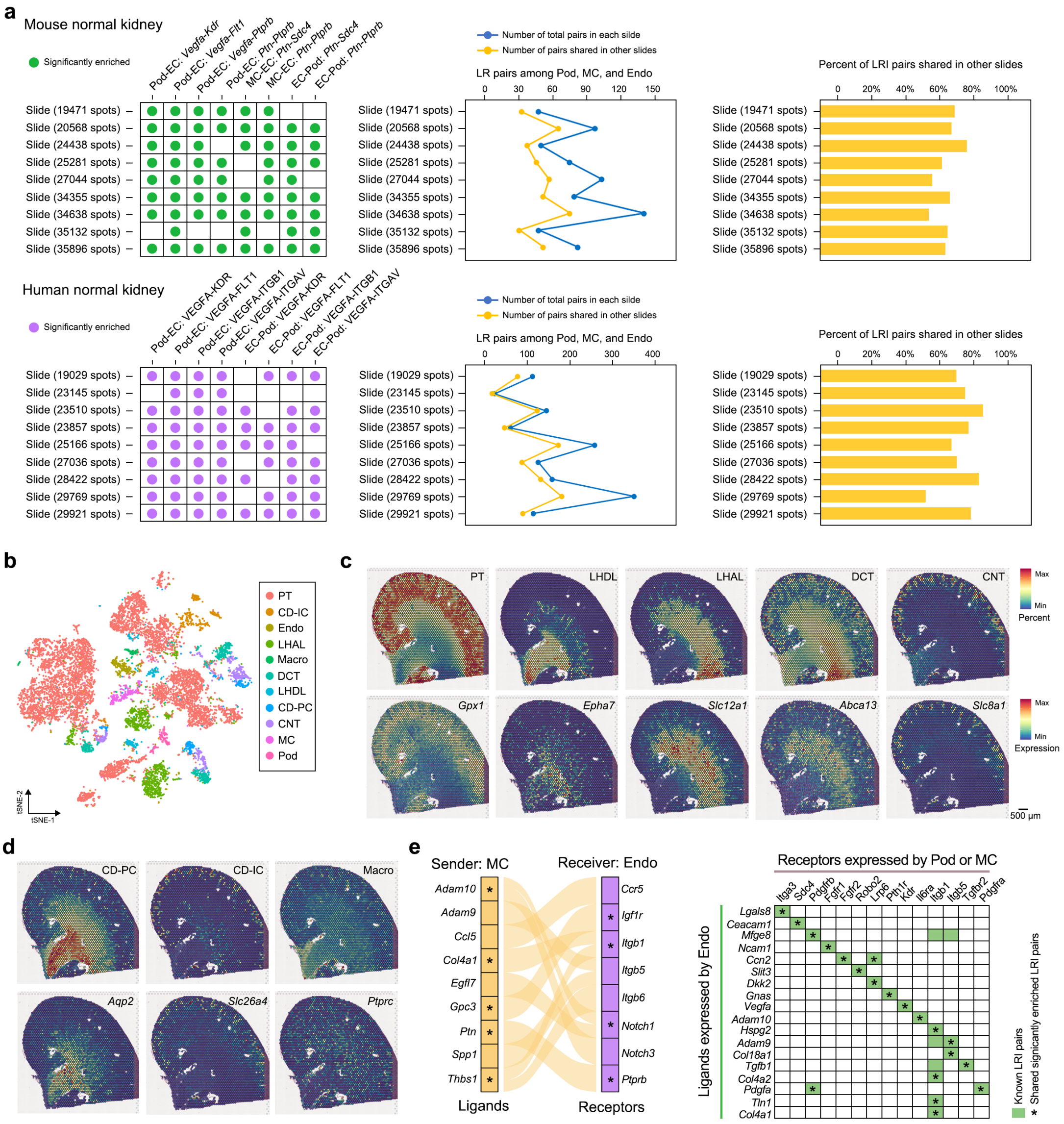

Fig. 5 Cell-cell communication inference among the glomerular cells in kidney by SpaTalk. a Inferred LRI pairs mediating the intraglomerular communications across different slides of mouse kidney sequenced by Slide-seq (v2). Shared LR pairs among different slides were plotted. **b** Inferred LRI pairs mediating the intraglomerular communications across different slides of human kidney sequenced by Slide-seq (v2). Shared LR pairs among different slides were plotted. **c** A previously published adult mouse kidney cell taxonomy by single-nucleus RNA-seq, which contains 11,395 single cells involving 11 cell types. CD-IC, collecting duct intercalated cells; CD-PC, collecting duct principal cells; DCT, distal convoluted tubules; Endo, endothelial cells; Macro, macrophages; MC, mesangial cells; PT, proximal tubules; Pod, podocytes; LHDL, loop of Henle descending loop; LHAL, loop of Henle ascending loop; CNT, connecting tubule. **d-e** Percent of PT, LHDL, LHAL, DCT, CNT, CD-PC, CD-IC, and Macro as well as the expression of the corresponding known markers. **f** Shared LRIs of mouse Visium ST data with that of Slide-seq (v2) inferred by SpaTalk. Cell-cell communication among MC, Pod, and Endo were plotted.

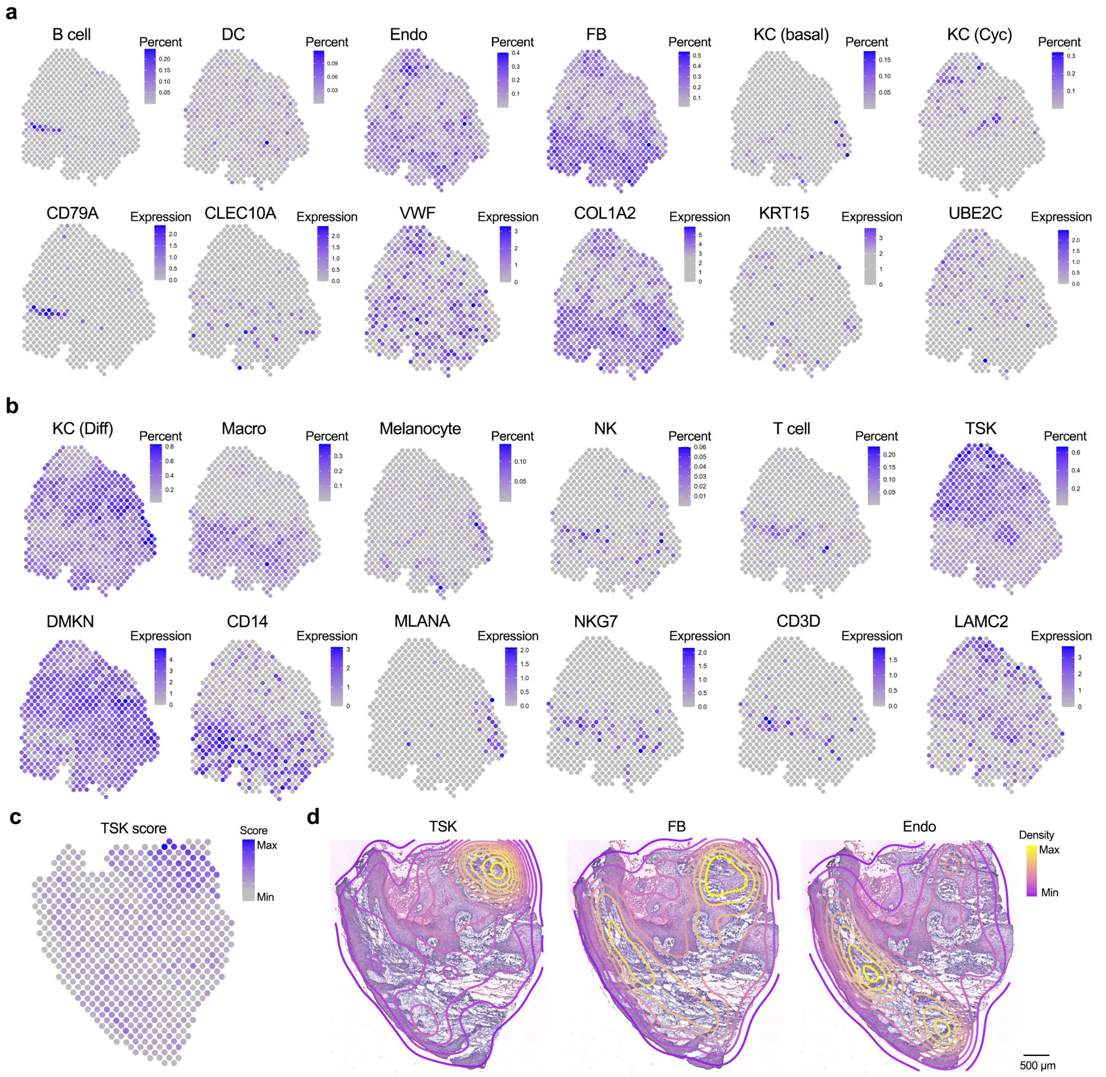

Fig. 6 Cell-type decomposition on the human skin SCC ST data of 10X Visium. **a-b** Expression of known marker gene (up) and the percent (down) for B cell, dendritic cell (DC), endothelial cell (Endo), fibroblast (FB), KC (basal), KC (Cycling), KC (Differentiating), macrophage (Macro), melanocyte, natural killer (NK) cell, T cell, and TSK. **c** TSK score across spatial spots using the signatures of TSK. **d** Contour plot of TSK, FB, and Endo based on the reconstructed single-cell ST atlas by SpaTalk in patient 10.

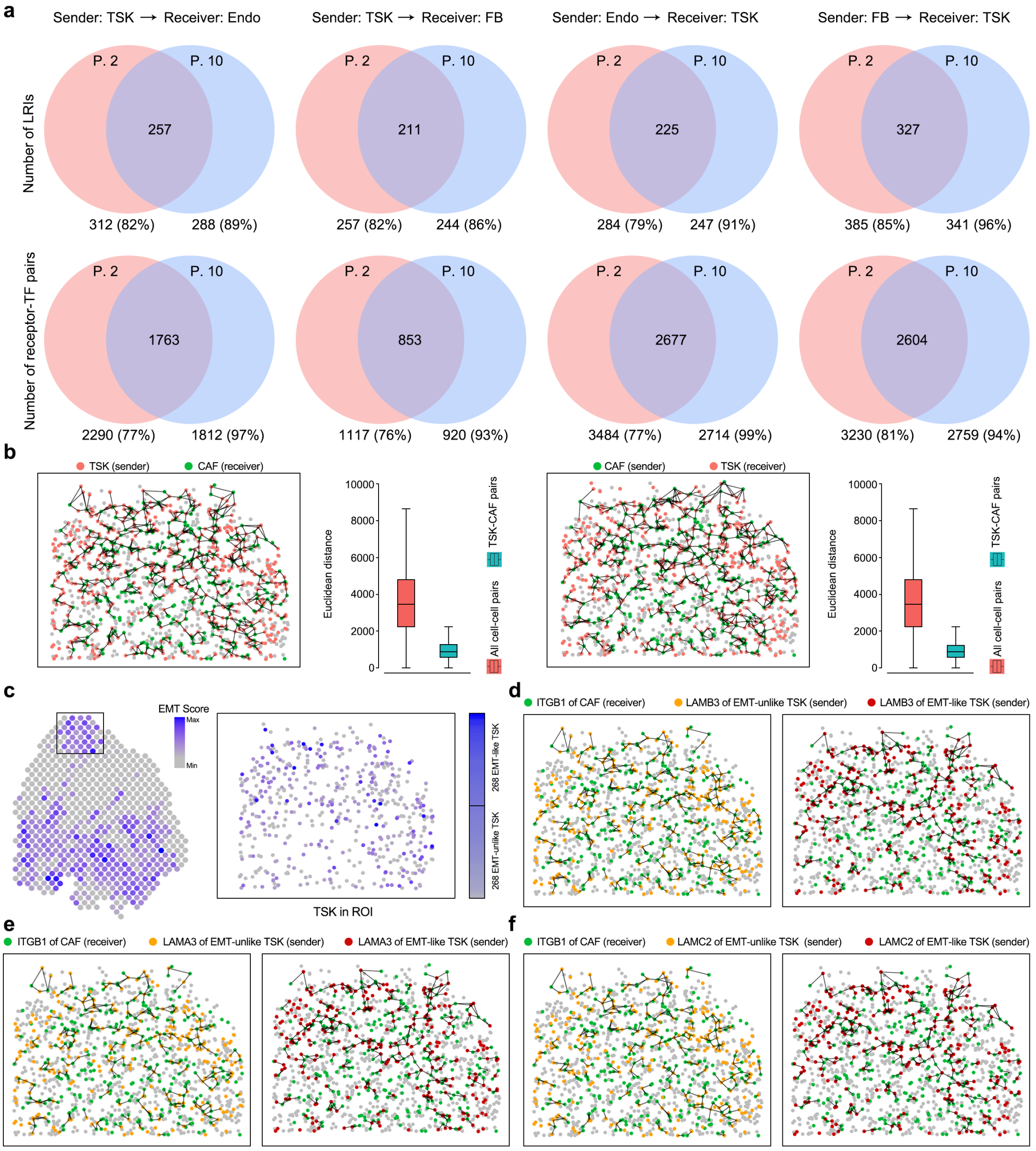

Fig. 7 Cell-cell communications between TSK subpopulation and stromal cells in space with SpaTalk. a Comparison of inferred LRI pairs and TFs by SpaTalk over 10X Visium ST datasets from two SCC patients (P. 2 and P. 10). The percent of shared LRI pairs and TFs for each patient was labeled beside the total number of inferred LRI pairs and TFs. **b** Direct cell-cell communication between TSK and CAF. Neighbor cells for TSK and CAF senders were plotted, respectively, wherein the left are the cell-cell pairs of TSK senders and CAF receivers and the right are the cell-cell pairs of CAF senders and TSK receivers. For the boxplots (minima, 25th percentile, median, 75th percentile, and maxima) of the red and green boxes, the numbers of data points are 966,735 and 1,035, respectively (left); the numbers of data points are 966,735 and 1,049 respectively (right). **c** EMT score across spatial spots using the signatures of EMT hallmarks, wherein TSKs were divided into EMT-like and EMT-unlike TSKs according to the EMT scores. **d-f** Number of cell-cell pairs from the EMT-like and EMT-unlike TSKs to CAFs over the LRIs, namely LAMB3-ITGB1, LAMA3-ITGB1, and LAMC2-ITGB1.

Supplementary Tables

Table 1. Computation time of the deconvolution step.

Methods	Simulated spot data				Real spot data
	STARmap	MERFISH	seqFISH OB	seqFISH SVZ	10X Visium
SpaTalk	0.13min	0.08min	2.18min	3.05min	6.23min
RCTD	1.35min	0.65min	1.20min	3.80min	3.33min
Seurat	0.22min	0.18min	0.90min	0.55min	2.47min
SPOTlight	1.68min	0.32min	4.30min	10.92min	26.83min
deconvSeq	2.18min	NA	25.85min	17.52min	NA
Stereoscope	56.58min	205.53min	359.20min	67.02min	604.58min
Cell2location	35.13min	20.30min	25.25min	30.02min	31.03min

NA, not available.

Table 2. Computation time of the inferring cell-cell communication step.

Methods	STARmap	seqFISH OB (FOV0)	seqFISH SVZ(FOV0)	10X Visium (TSK-Endo)
SpaTalk	0.47min	3.40min	22.05min	4.72min
Giotto	5.02min	5.06min	3.99min	37.11min
SpaOTsc	0.08min	0.09min	0.11min	26.70min
NicheNet	19.39min	13.09min	11.00min	4.59min
CytoTalk	42.03min	>12h	>12h	123.68min
CellCall	2.34min	12.66min	16.26min	14.86min
CellPhoneDB	1.57min	1.67min	3.69min	11.30min
CellChat	0.69min	67.65min	123.90min	41.48min

Table 3. Biological processes of GO analysis of TSK receptors.

Gene	GO ID	GO term
PTPRZ1	GO:0007417	central nervous system development
PTPRZ1	GO:0002244	hematopoietic progenitor cell differentiation
PTPRZ1	GO:0048709	oligodendrocyte differentiation
PTPRZ1	GO:0048714	positive regulation of oligodendrocyte differentiation
PTPRZ1	GO:0070445	regulation of oligodendrocyte progenitor proliferation
SDC1	GO:0060070	canonical Wnt signaling pathway
SDC1	GO:0016477	cell migration
SDC1	GO:0050900	leukocyte migration
SDC1	GO:0048627	myoblast development
SDC1	GO:0060009	Sertoli cell development
SDC1	GO:0055002	striated muscle cell development
SDC1	GO:0001657	ureteric bud development
ITGA6	GO:0098609	cell-cell adhesion
ITGA6	GO:0007160	cell-matrix adhesion
ITGA6	GO:0050900	leukocyte migration
ITGA6	GO:0050873	brown fat cell differentiation
ITGA6	GO:0010668	ectodermal cell differentiation
ITGA6	GO:0030198	extracellular matrix organization
ITGA6	GO:0022409	positive regulation of cell-cell adhesion
ITGA6	GO:0030335	positive regulation of cell migration
ITGA6	GO:0035878	nail development
