# Supplementary material for: Knowledge-graph-based cell-cell communication inference for spatially resolved transcriptomic data with SpaTalk
Source: Nat Commun. 2022 Jul 30;13:4429. doi: 10.1038/s41467-022-32111-8 (PMC9338929; doi:10.1038/s41467-022-32111-8)
Supplement: Supplementary file 3 — Reporting Summary [file 41467_2022_32111_MOESM3_ESM.pdf]

nature portfolio

Corresponding author(s): Xiaohui Fan

Last updated by author(s): Jul 8, 2022

Reporting Summary

Nature Portfolio wishes to improve the reproducibility of the work that we publish. This form provides structure for consistency and transparency in reporting. For further information on Nature Portfolio policies, see our Editorial Policies and the Editorial Policy Checklist.

Statistics

For all statistical analyses, confirm that the following items are present in the figure legend, table legend, main text, or Methods section.

n/a	Confirmed	
☐	☒	The exact sample size (*n*) for each experimental group/condition, given as a discrete number and unit of measurement
☒	☐	A statement on whether measurements were taken from distinct samples or whether the same sample was measured repeatedly
☐	☒	The statistical test(s) used AND whether they are one- or two-sided<br>*Only common tests should be described solely by name; describe more complex techniques in the Methods section.*
☒	☐	A description of all covariates tested
☐	☒	A description of any assumptions or corrections, such as tests of normality and adjustment for multiple comparisons
☐	☒	A full description of the statistical parameters including central tendency (e.g. means) or other basic estimates (e.g. regression coefficient) AND variation (e.g. standard deviation) or associated estimates of uncertainty (e.g. confidence intervals)
☐	☒	For null hypothesis testing, the test statistic (e.g. *F*, *t*, *r*) with confidence intervals, effect sizes, degrees of freedom and *P* value noted<br>*Give P values as exact values whenever suitable.*
☒	☐	For Bayesian analysis, information on the choice of priors and Markov chain Monte Carlo settings
☒	☐	For hierarchical and complex designs, identification of the appropriate level for tests and full reporting of outcomes
☐	☒	Estimates of effect sizes (e.g. Cohen's *d*, Pearson's *r*), indicating how they were calculated

Our web collection on statistics for biologists contains articles on many of the points above.

Software and code

Policy information about availability of computer code

Data collection	The STARmap software (https://github.com/weallen/STARmap) was used to collect the mouse visual cortex spatial data. Other data were collected by downloading the raw data directly from public platforms.
Data analysis	Data processing was performed by R 4.1.1, Python 3.9, and GraphPad Prism 8. Pathway enrichment analysis was performed by Metascape web tool (https://metascape.org/). All related codes were uploaded to the Github (https://github.com/ZJUFanLab/SpaTalk)

For manuscripts utilizing custom algorithms or software that are central to the research but not yet described in published literature, software must be made available to editors and reviewers. We strongly encourage code deposition in a community repository (e.g. GitHub). See the Nature Portfolio guidelines for submitting code & software for further information.

Data

Policy information about availability of data

All manuscripts must include a data availability statement. This statement should provide the following information, where applicable:

- Accession codes, unique identifiers, or web links for publicly available datasets
- A description of any restrictions on data availability
- For clinical datasets or third party data, please ensure that the statement adheres to our policy

The original data used in this paper can be accessed through the following links: (1) STARmap spatial data of the mouse cortex (https://www.dropbox.com/sh/f7ebheru1lbz91s/AABYSSjSTppBmVmWl2H4sKa?dl=0); (2) MERFISH data of the naïve female mouse hypothalamic preoptic region (https://datadryad.org/stash/dataset/doi:10.5061/dryad.8t8s248); (3) seqFISH+ data of the mouse cortex and olfactory bulb: downloaded from Github repository (https://github.com/CaiGroup/seqFISH-PLUS); (4) Slide-seq data of the mouse liver (https://singlecell.broadinstitute.org/single_cell/study/SCP354/slide-seq-study), somatosensory cortex (https://singlecell.broadinstitute.org/single_cell/study/SCP815/highly-sensitive-spatial-transcriptomics-at-near-cellular-resolution-with-slide-seqv2), and the human and

mouse kidney (https://cellxgene.cziscience.com/collections/8e880741-bf9a-4c8e-9227-934204631d2a); (5) spatial data and scRNA-seq data of human SCC: GEO accession: GSE144240 (https://www.ncbi.nlm.nih.gov/geo/query/acc.cgi?acc=GSE144240); (6) spatial data of the mouse kidney: downloaded from 10X Visium Spatial Gene Expression (https://www.10xgenomics.com/resources/datasets); (7) Single-nucleus RNA-sequencing data of the mouse kidney: GEO accession: GSE119531 (https://www.ncbi.nlm.nih.gov/geo/query/acc.cgi?acc=GSE119531); (8) mouse liver scRNA-seq data of non-parenchymal cells (https://figshare.com/articles/MCA_DGE_Data/5435866) and parenchymal hepatic cells: GEO accession: GSE125688 (https://www.ncbi.nlm.nih.gov/geo/query/acc.cgi?acc=GSE125688); (9) mouse cortex scRNA-seq data: GEO accession: GSE71585 (https://www.ncbi.nlm.nih.gov/geo/query/acc.cgi?acc=GSE71585). Molecular Signatures Database was downloaded from MSigDB v7.4 (http://www.gsea-msigdb.org/gsea/msigdb). Source data are provided with this paper.

Field-specific reporting

Please select the one below that is the best fit for your research. If you are not sure, read the appropriate sections before making your selection.

[x] Life sciences   [ ] Behavioural & social sciences   [ ] Ecological, evolutionary & environmental sciences

For a reference copy of the document with all sections, see nature.com/documents/nr-reporting-summary-flat.pdf

Life sciences study design

All studies must disclose on these points even when the disclosure is negative.

Sample size	No sample-size estimates were performed. We collected all accessible datasets covering different tissue types to demonstrate and compare the performance.
Data exclusions	No data was excluded. Filtering and quality control of single cells is described in the Methods.
Replication	All the computational experiments were replicated four times (twice on two Windows computers and twice on two Ubuntu 18.04 servers). All attempts at replication were successful.
Randomization	The allocation was random involving the computational algorithms.
Blinding	No new data was generated for this study. All results are based on published data which have been studied in their original publications. Therefore, blinding from investigators is not possible when we reanalyzed the collected data. The computational algorithm do not include the training step, hence all samples were used to generate the unbiased results without group allocation. Hyperparameters were set to commonly used values, which can produce robust results under different hyperparameters.

Reporting for specific materials, systems and methods

We require information from authors about some types of materials, experimental systems and methods used in many studies. Here, indicate whether each material, system or method listed is relevant to your study. If you are not sure if a list item applies to your research, read the appropriate section before selecting a response.

Materials & experimental systems

n/a	Involved in the study	
[x]	[ ]	Antibodies
[x]	[ ]	Eukaryotic cell lines
[x]	[ ]	Palaeontology and archaeology
[x]	[ ]	Animals and other organisms
[x]	[ ]	Human research participants
[x]	[ ]	Clinical data
[x]	[ ]	Dual use research of concern

Methods

n/a	Involved in the study	
[x]	[ ]	ChIP-seq
[x]	[ ]	Flow cytometry
[x]	[ ]	MRI-based neuroimaging
